# A multicenter study of antimicrobial prescriptions for cats diagnosed with bacterial urinary tract disease

**DOI:** 10.1177/1098612X211054815

**Published:** 2021-10-28

**Authors:** J Scott Weese, Jason W Stull, Michelle Evason, Jinelle Webb, Dennis Ballance, Talon McKee, Philip J Bergman

**Affiliations:** 1 Ontario Veterinary College, University of Guelph, Guelph, ON, Canada; 2 Atlantic Veterinary College, University of Prince Edward Island, Charlottetown, PE, Canada; 3 Mississauga-Oakville Veterinary Emergency and Specialty Hospital, Oakville, ON, Canada; 4 VCA Clinical Studies, Los Angeles, CA, USA

**Keywords:** Antimicrobials, antimicrobial stewardship, urinary tract disease, antimicrobial resistance

## Abstract

**Objectives:**

The aim of this study was to evaluate initial antimicrobial therapy in cats diagnosed with upper or lower bacterial urinary tract infections at veterinary practices in the USA and Canada.

**Methods:**

Electronic medical records from a veterinary practice corporation with clinics in the USA and Canada were queried between 2 January 2016 and 3 December 2018. Feline patient visits with a diagnosis field entry of urinary tract infection, cystitis and pyelonephritis, as well as variation of those names and more colloquial diagnoses such as kidney and bladder infection, and where an antimicrobial was prescribed, were retrieved.

**Results:**

Prescription data for 5724 visits were identified. Sporadic cystitis was the most common diagnosis (n = 5051 [88%]), with 491 (8.6%) cats diagnosed with pyelonephritis and 182 (3.2%) with chronic or recurrent cystitis. Cefovecin was the most commonly prescribed antimicrobial for all conditions, followed by amoxicillin–clavulanic acid. Significant differences in antimicrobial drug class prescribing were noted between practice types and countries, and over the 3-year study period. For sporadic cystitis, prescription of amoxicillin–clavulanic acid increased significantly and cefovecin decreased between 2016 and 2018, and 2017 and 2018, while fluoroquinolone use increased between 2017 and 2018.

**Conclusions and relevance:**

The results indicate targets for intervention and some encouraging trends. Understanding how antimicrobials are used is a key component of antimicrobial stewardship and is required to establish benchmarks, identify areas for improvement, aid in the development of interventions and evaluate the impact of interventions or other changes.

## Introduction

Infectious urinary tract disease is commonly diagnosed in cats, with resultant common use of antimicrobials.^1^ Antimicrobial stewardship aims to optimize antimicrobial therapy in patients, maximizing clinical outcomes, while minimizing adverse effects on the patient (eg, gastro-intestinal complications) or population (eg, selection pressure for antimicrobial resistance). A core aspect of antimicrobial stewardship is understanding how antimicrobials are used, as this is required to assess anti-microbial use practices, establish benchmarks and targets, identify areas for improvement, develop interventions, and evaluate the impact of interventions or other changes.

The objective of this multicenter study was to evaluate initial antimicrobial therapy in cats diagnosed with upper or lower bacterial urinary tract infections at a subset of veterinary practices in the USA and Canada.

## Materials and methods

Electronic medical records from a veterinary practice corporation with clinics in the USA and Canada were queried between 2 January 2016 and 3 December 2018. Feline patient visits with a diagnosis field entry of urinary tract infection, cystitis and pyelonephritis, as well as variation of those names and more colloquial diagnoses such as kidney and bladder infection, and where one or more antimicrobial was prescribed, were retrieved. Based on free-text entries in the clinical diagnosis field code, cases were classified by one author (JSW) into sporadic bacterial cystitis, chronic or recurrent cystitis (referred to herein as recurrent cystitis), pyelonephritis and other, with no further study of the ‘other’ group. Because there were numerous descriptions in the open-entry field, a subjective determination for classification was required in some cases. This was performed while blinded to other data fields (eg, drug and duration). ‘Suspect’ or ‘possible’ entries were included because they were accompanied by an antimicrobial prescription for that condition. When entries indicated the potential presence of a comorbidity that might impact antimicrobial decision-making (eg, wound infection and dermatitis) the record was removed. A repeat visit of the same animal within 30 days was removed unless there was a different field code (eg, initial diagnosis of cystitis and then subsequent visit diagnosis as pyelonephritis), on the assumption that a new treatment decision would have been made for the second disease occurrence.

Signalment, body weight, practice location (zip code or postal code), visit date, diagnosis and antimicrobial drug regimens were recorded. Duration of treatment was determined based on specific prescription recommendations in the record (eg, administer for 14 days), or, when that was not specified, calculated based on the recommended dose (eg, ‘give two tablets twice daily’) and the amount of drug that was dispensed. If inadequate detail was present to accurately determine duration, the duration field was left blank, but records were retained for analysis of drug selection. Each dose of cefovecin was considered to be a 14-day treatment duration.^2^

The antimicrobial(s) prescribed and duration of treatment were the outcomes of interest. Antimicrobial use analysis was performed at the drug-class level (eg, penicillins and potentiated penicillins). Antimicrobials were also categorized as per World Health Organization criteria,^3^ assigning antimicrobials to highest priority, critically important antimicrobial (HP-CIA); critically important antimicrobial (CIA); and highly important antimicrobial (HIA) groups. Prescription practices were compared with 2011 International Society for Companion Animal Infectious Diseases (ISCAID) guidelines.^4^ For sporadic bacterial cystitis, two analyses were performed. One considered amoxicillin and potentiated sulfonamides as first-line recommendations, while the second added amoxicillin–clavulanic acid, based on the guideline statement that the drug is ‘acceptable’.

Continuous data were evaluated for normality using Shapiro–Wilk (for sample sizes ⩽2000) or Kolmogorov–Smirnov test with Lilliefors correction tests (for sample sizes >2000). Non-normally distributed data were reported as median and interquartile range (IQR). Categorical data were assessed using Fisher’s exact or χ^2^ tests (based on associated assumptions and minimum expected cell values), while continuous data were analyzed using Wilcoxon, Steel or Steel–Dwass tests. Odds ratios (OR) and accompanying 95% confidence intervals (95% CI) were calculated. Data were analyzed using JMP15 (SAS Institute). For all analyses, *P* <0.05 was considered significant.

## Results

Prescription data for 5724 visits were identified. Data were from 610 clinics: 561 (92%) from the USA and 49 (8.0%) from Canada. Most cat visits (n = 5028 [88%]) were to general practices, while 301 (5.3%) were to mixed general and specialty practices (referred to henceforth as ‘combined clinics’), 218 (3.8%) were to specialty practices, 103 (1.8%) were to feline-only practices and 74 (1.3%) were to emergency clinics. Feline-only and specialty clinics were only represented in 2017 and 2018. Most (n = 5515 [96%]) visit records were from the USA, while 209 (3.6%) were from Canada.

Sporadic cystitis was the most common diagnosis (n = 5051 [88%]), with 491 (8.6%) cats diagnosed with pyelonephritis and 182 (3.2%) with chronic or recurrent cystitis. In 2016, 1246 (22%) cat visits resulted in the prescription of antimicrobials, which increased to 2226 (39%) in 2017 and 2252 (39%) in 2018. A total of 6112 individual antimicrobial prescriptions were identified (median 1/visit; range 1–3). Overall, 5370 (93.8%) of cats were prescribed a single antimicrobial, while combination therapy was prescribed to 354 (6.2%). One or more drugs in the HP-CIA class were prescribed to 4319 (75%) of cats. The most commonly prescribed antimicrobials and combinations are presented in Table 1.

**Table 1:** Most commonly prescribed antimicrobials or antimicrobial combinations for 5724 cats diagnosed with sporadic cystitis, recurrent cystitis and pyelonephritis

Sporadic cystitis (n = 5051)	Recurrent cystitis (n = 182)	Pyelonephritis (n = 491)	Overall (n = 5724)
Cefovecin* (n = 3056 [61%])	Cefovecin* (n = 110 [60%])	Cefovecin* (n = 132 [27%])	Cefovecin* (n = 3298 [58%])
Amoxicillin–clavulanic acid^†^ (n = 1076 [21%])	Amoxicillin–clavulanic acid^†^ (n = 25 [14%])	Amoxicillin–clavulanic acid^†^ (n = 90 [18%])	Amoxicillin–clavulanic acid^†^ (n = 1191 [21%])
Orbifloxacin* (n = 203 [4.0%])	Marbofloxacin* (n = 14 [7.7%])	Marbofloxacin* (n = 82 [17%])	Marbofloxacin* (n = 285 [5.0%])
Marbofloxacin* (n = 189 [3.7%])	Orbifloxacin* (n = 13 [7.1%])	Orbifloxacin* (n = 32 [6.5%])	Orbifloxacin* (n = 248 [4.3%])
Amoxicillin^‡^ (n = 164 [3.2%])Cefovecin* + amoxicillin–clavulanic acid^†^ (n = 123 [2.4%])	Amoxicillin^‡^ (n = 8 [4.4%])Enrofloxacin* (n = 4 [2.2%])	Amoxicillin–clavulanic acid^†^ + marbofloxacin* (n = 22 [4.5%])	Amoxicillin^‡^ (n = 183 [3.2%])Cefovecin* + amoxicillin–clavulanic acid^†^ (n = 130 [2.3%])
		Enrofloxacin* (n = 21 [4.3%])	
Enrofloxacin* (n = 44 [0.9%])Cefovecin* + marbofloxacin* (n = 28 [0.6%])	Cefovecin* + amoxicillin–clavulanic acid^†^ (n = 2 [1.1%])	Pradofloxacin* (n = 18 [3.7%])Cefovecin* + marbofloxacin* (n = 16 [3.3%])	Enrofloxacin* (n = 69 [1.2%])Cefovecin* + marbofloxacin* (n = 45 [0.8%])
	Pradofloxacin* (n = 2 [1.1%])		

* World Health Organization (WHO) antimicrobial classification: highest priority critically important

† WHO antimicrobial classification: critically important

‡ WHO antimicrobial classification: highly important

### Sporadic bacterial cystitis

There were 4873 (96%) cats from the USA and 178 (3.5%) from Canada with sporadic bacterial cystitis, from general practices (n = 4525 [90%]), combined clinics (n = 243 [4.8%]), specialty clinics (n = 157 [3.1%]), emergency clinics (n = 72 [1.4%]) and feline-only clinics (n = 54 [1.1%]). The median age of the cats was 8.0 years (IQR 9.2). Most (n = 2811 [56%]) were spayed females, 1968 (39%) were castrated males, 82 (1.6%) were intact females and 68 (1.3%) were intact males. Sex was not reported for 122 cats (2.4%). One thousand and ninety-three (22%) cats were from 2016, 1953 (39%) were from 2017 and 2005 (40%) were from 2018.

**Figure 1:**
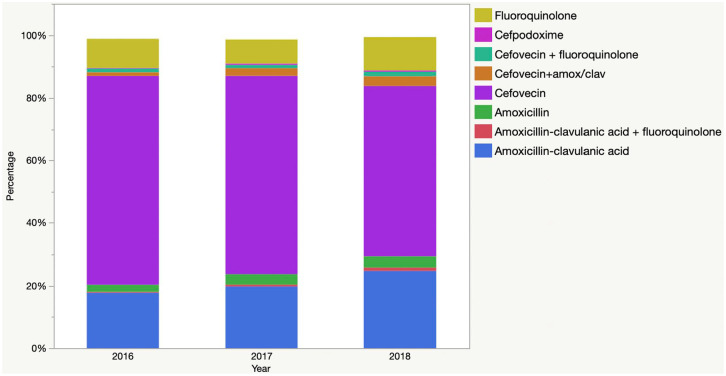
Antimicrobial classes prescribed to 5051 cats diagnosed with sporadic bacterial cystitis, by year

A total of 15 antimicrobials were prescribed; however, only cefovecin, amoxicillin–clavulanic acid, orbifloxacin, marbofloxacin and enrofloxacin accounted for at least 1% of prescriptions each. The most commonly prescribed antimicrobials and combinations are presented in Table 1 and Figure 1. Most cats (n = 4796 [95%]) were prescribed a single antimicrobial, while 255 (5%) were prescribed more than one. Two hundred and three of those (80%) received cefovecin plus an oral antimicrobial, corresponding to 4% of cats overall. One or more drug(s) from the HP-CIA class was prescribed to 3789 (75%) of cats.

**Figure 2:**
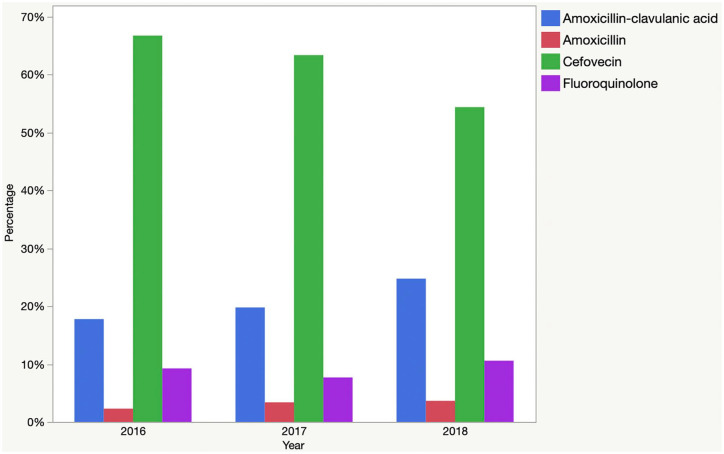
Percentage of cats diagnosed with sporadic bacterial cystitis (n = 5051) prescribed the main antimicrobials, alone or as part of a combination, as well as the percentage of cats receiving at least one drug classified as a highest priority critically important antimicrobial (HP-CIA) by the World Health Organization

There were significant inter-year differences in the relative use of amoxicillin, amoxicillin–clavulanic acid, cefovecin and fluoroquinolones, as well as overall use of HP-CIAs (Figure 2, Table 2).

**Table 2:** Odds ratios (ORs), 95% confidence intervals (CIs) and *P* values for the prescription of antimicrobial drugs, classes or grouping for sporadic bacterial cystitis in 5051 feline visits

Antimicrobial class/group and year	n (%)	2016 vs 2017		2016 vs 2018		2017 vs 2018	
		OR (95% CI)	*P* value	OR (95% CI)	*P* value	OR (95% CI)	*P* value
Amoxicillin		1.32 (0.85–2.03)	0.21	1.43 (0.94–2.20)	0.10	1.10 (0.78–1.50)	0.61
2016	30/1093 (2.7)						
2017	70/1953 (3.6)						
2018	78/2005 (3.9)						
Amoxicillin–clavulanic acid		1.10 (0.92–1.31)	0.30	1.50 (1.26–1.79)	<0.0001	1.37 (1.18–1.58)	<0.0001
2016	234/1093 (21)						
2017	450/1953 (23)						
2018	582/2005 (29)						
Fluoroquinolones		0.85 (0.66–1.08)	0.18	1.20 (0.96–1.51)	0.11	1.41 (1.16–1.73)	0.0006
2016	122/1093 (11)						
2017	188/1953 (9.6)						
2018	263/2004 (13)						
Cefovecin		0.91 (0.77–1.06)	0.23	0.64 (0.54–0.74)	<0.0001	0.70 (0.62–0.80)	<0.0001
2016	760/1093 (70)						
2017	1317/1953 (67)						
2018	1187/2005 (59)						
HP-CIA		0.82 (0.69–0.99)	0.034	0.64 (0.53–0.76)	<0.0001	0.77 (0.67–0.89)	0.0005
2016	870/1093 (80)						
2017	1489/1953 (76)						
2018	1430/2005 (71)						

OR referent is the earlier year of the comparison; OR >1 signifies an increase in prescribing of the given antimicrobial class/group in the later year (vs earlier year), while an OR <1 signifies a decrease in prescribing

HP-CIA = highest priority critically important antimicrobial

**Figure 3:**
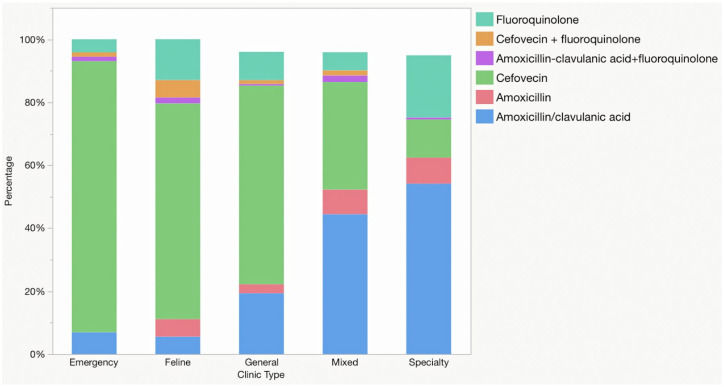
Antimicrobial classes prescribed to 5051 cats diagnosed with sporadic bacterial cystitis, by clinic type

Prescription of the most common antimicrobials by clinic type is presented in Figure 3. There were significant differences among clinic types in the use of amoxicillin–clavulanic acid (*P* <0.0001), amoxicillin (*P* <0.0001), fluoroquinolones (*P* = 0.0002) and cefovecin (*P* <0.0001). HP-CIAs were prescribed during 36% (n = 57/157) of cat visits at specialty practices, 47% (n = 114/243) at combined practices, 77% (n = 3503/4525) at general practices, 89% (n = 48/54) at feline-only clinics and 93% (n = 67/72) at emergency clinics (*P* <0.0001)

National comparison (USA vs Canada) was only performed using general practices, owing to small sample sizes of the other clinic types. In the USA, there was significantly more prescriptions of cefovecin (OR 2.6, 95% CI 1.9–3.6; *P* <0.0001) and less of amoxicillin–clavulanic acid (OR 0.25, 95% CI 0.18–0.35; *P* <0.0001) compared with Canadian practices, with no difference in amoxicillin (*P* = 0.40) or fluoroquinolones (*P* = 0.21). Prescription of HP-CIAs was more common in the USA than in Canadian practices (OR 3.3, 95% CI 2.4–4.6; *P* <0.0001).

The median duration of prescribed therapy was 14 days (IQR 0) overall, and for each year. There was no impact of visit year on duration (*P* = 0.13) overall. Prescriptions for cefovecin were of a significantly longer duration compared with all other prescriptions (14 days vs 10 days; *P* <0.001). When cefovecin prescriptions are excluded, there was a non-significant trend to decreased duration in 2018 vs 2017 (both median 10 days; *P* = 0.067)

There was a significant difference in duration between specialty clinics (10 days) and combined (14 days, *P* = 0.014), feline (14 days, *P* <0.001), emergency (14 days, *P* <0.001) and general (14 days, *P* <0.001) clinics. There was also a significantly shorter treatment duration for combined clinics compared with emergency (*P* = 0.003), feline (*P* = 0.003) and general practices (*P* <0.001).

There was a significantly shorter prescribed duration in Canada (median 10 days; IQR 4) than the USA (median 14 days; IQR 0) (*P* = 0.0079).

Only 56 (1.1%) of prescriptions were consistent with the 2011 ISCAID guidelines (7 days of treatment with amoxicillin or trimethoprim–sulfonamide).^5^ If amoxicillin–clavulanic was included as a first-line treatment, this increased to 434 (8.6%). There was increased consistency with the 2011 guidelines over the study period (*P* = 0.005), increasing from 0.64% (n = 7/1093) in 2016 to 1.2% in both 2017 (n = 24/1953) and 2018 (n = 25/2005).

**Figure 4:**
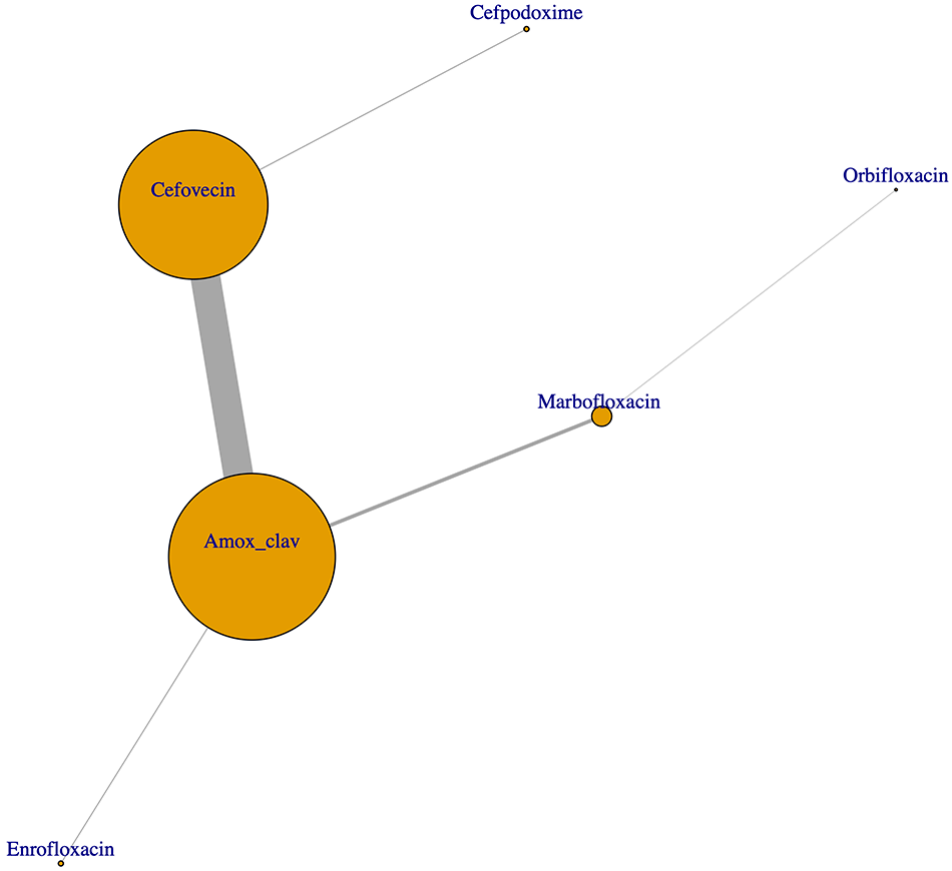
Network analysis depicting co-prescription of antimicrobials to cats diagnosed with sporadic bacterial cystitis

Network analysis, indicating antimicrobial combination relationships, is presented in Figure 4.

### Pyelonephritis

There were 468 (95%) cases of pyelonephritis from the USA and 23 (4.7%) from Canada, from general practices (n = 333 [68%]), specialty clinics (n = 57 [12%]), combined clinics (n = 53 [11%]), feline-only clinics (n = 46 [9.4%]) and emergency clinics (n = 2 [0.4%]). Owing to the small sample size, emergency clinics were excluded from analysis of the impact of clinic type.

The median age of the cats was 13.7 years (IQR 6). Most (n = 319 [65%]) were spayed females, 157 (32%) were castrated males, five (1.0%) were intact males and five (1.0%) were intact females. Sex was not reported for five (1.0%) cats. One hundred (20%) cases were from 2016, 204 (42%) were from 2017 and 187 (38%) were from 2018.

Forty-nine percent (n = 243/491) of cats were prescribed a fluoroquinolone, alone or in combination with another antimicrobial, while 34% (n = 169/491) of cats were prescribed cefovecin. The most commonly prescribed antimicrobials and combinations are presented in Table 1. Nineteen percent (n = 95/491) of cats were prescribed more than one antimicrobial, the most common combination being cefovecin and amoxicillin–clavulanic acid. Thirty-six (7.3%) cats received an oral antimicrobial in conjunction with injectable cefovecin. Most cats (n = 383 [78%]) were prescribed one or more HP-CIAs.

**Figure 5:**
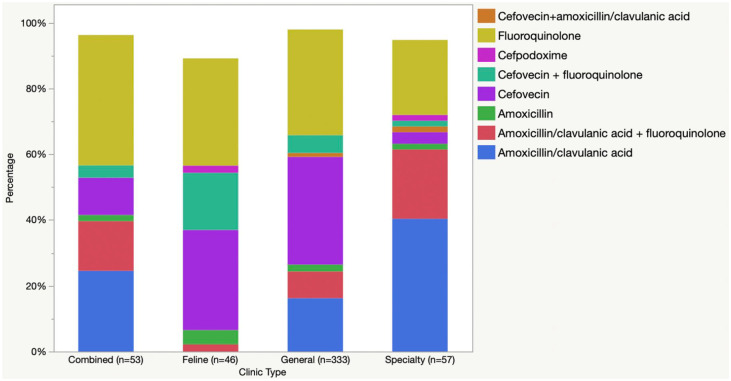
Antimicrobial classes prescribed to 491 cats diagnosed with pyelonephritis, by clinic type

There was an impact of clinic type on the use of amoxicillin–clavulanic acid (*P* <0.0001) and cefovecin (*P* <0.0001) (Figure 5). Cefovecin, alone or in combination, was prescribed to 7.0% (n = 4/57) of cats at specialty clinics, 15% (n = 8/53) at combined clinics, 40% (n = 133/333) at general practices and 50% (n = 23/46) at feline-only clinics. There was also an association between clinic type and HP-CIA prescription, with the prescription ranging from 56% (n = 32/57) at specialty clinics to 91% (n = 42/46) at feline-only clinics (*P* <0.001). There was a significant decrease in prescription of cefovecin between 2016 and 2018 (OR 0.55, 95% CI 0.33–0.92; *P* = 0.021) and a significant increase in amoxicillin–clavulanic acid use between 2017 and 2018 (OR 1.7, 95% CI 1.1–2.6; *P* = 0.020). There were no other significant differences between the use of the main drug classes or HP-CIAs over time.

The median duration of treatment was 14 days (IQR 14). This did not change from 2016 to 2018 (*P* = 0.62). There was no identifiable association between duration and clinic type (all *P* >0.25) or country (*P* = 0.14).

Ten percent (n = 49/491) of prescriptions were consistent with the 2011 ISCAID recommendations of 4–6 weeks for a fluoroquinolone. This did not vary with year (*P* = 0.16) or clinic type (*P* = 0.19)

### Recurrent cystitis

Recurrent cystitis was seen in 174 (95.6%) cats from the USA and eight (4.4%) from Canada. One hundred and seventy (93.4%) were from general practices, five (2.8%) from combined clinics, four (2.2%) from specialty clinics and three (1.7%) from feline-only clinics. The median age of the cats was 10.5 years (IQR 9.8). Most (n = 119 [65%]) were spayed females, 53 (29%) were castrated males, two (1.1%) were intact females and two (1.1%) were intact males. Sex was not reported for six (3.3%) cats. Fifty-three (29%) cases were from 2016, 69 (38%) were from 2017 and 60 (33%) were from 2018. Analyses involving country or clinic type were not performed because of the small sample sizes.

The most common drugs and combinations are presented in Table 1. Sixty-three percent (n = 114/182) of cats were prescribed cefovecin, alone or in combination with another drug, while 19% (n = 35/182) of cats were prescribed a fluoroquinolone. Correspondingly, 81% (n = 147/182) of cats were prescribed one or more HP-CIAs.

The median prescribed duration was 14 days (IQR 0, range 7–84). There was no impact of visit year (*P* = 0.22), clinic type (all *P* >0.26) or country (*P* = 0.087) on duration.

## Discussion

While clear targets for prescribing improvements and stewardship efforts were apparent, such as the duration of treatment and frequent use of second-tier drugs (including very frequent use of HP-CIAs), this study identifies both areas for intervention and some positive indicators, particularly for the treatment of sporadic cystitis. These positive indicators include increasing consistency with ISCAID guidelines over the 3-year study period, with less use of third-generation cephalosporins and fluoroquinolones, and a suggestion of decreasing duration of treatment. These changes were of relatively low magnitude, so, while encouraging, there is still a need for interventions pertaining to drug selection and treatment duration. Comparison with ISCAID guidelines is interesting but must done cautiously as patient-level considerations could not be assessed (eg, inability to medicate orally and lack of linked culture data). ISCAID guidelines are also largely expert opinion-based and, while useful, they do not necessarily reflect optimal treatments and some recommendations have changed (eg, duration of treatment) between the 2011 guidelines that were relevant at the time of this study and the subsequent 2019 guidelines. Regardless, there were clear differences in the prescription patterns noted here compared with the recommended first-line antimicrobials.

Frequent use of cefovecin was not surprising, as the ease of use of this single-injection treatment is particularly appealing for cat owners. Yet, as a third-generation cephalosporin that is expected to provide 14 days of therapeutic effect in urine, it is broader spectrum and longer in duration than is needed for treatment of acute cystitis. The need for its use could not be investigated because reasons for drug choice (eg, fractious cat) were not available in the medical record. Frequent use of this drug has been reported in other studies. In studies of primary care veterinary clinics in Canada, the UK and Australia, cefovecin accounted for 15–54% of antimicrobial prescriptions.^1,6–9^ Similarly, cefovecin accounted for 10–26% of prescription for urinary tract disease.^1,10^ It is reasonable to suspect that a long-acting injectable drug such as cefovecin was often not necessary, but different approaches are needed to study that. Additionally, there are efficacy concerns associated with use of cefovecin for pyelonephritis. While cefovecin achieves high levels in urine, pyelonephritis is an infection of renal tissue, not urine. As a highly protein-bound drug, there is limited active free drug in tissue (including renal parenchyma). There are no Clinical and Laboratory Standards Institute (CLSI) breakpoints for Enterobacterales in tissue, because of the questionable ability of cefovecin to reach effective concentrations for Enterobacterales in tissue. Since Enterobacterales, particularly *Escherichia coli*, are the most commonly diagnosed causes of pyelonephritis, cefovecin is not an appropriate choice for pyelonephritis. While the decrease in use of cefovecin for pyelonephritis during the study period was encouraging, there was a concurrent increase in use of amoxicillin–clavulanic acid monotherapy, something that is also of concern. While potentiated penicillins are excellent for bacterial cystitis because of the high drug levels in urine, CLSI guidelines^11^ indicate that Enterobacterales should be reported as resistant to amoxicillin–clavulanic acid for infections outside of the lower urinary tract because inadequate drug levels are achieved in tissue.

Consistent with the results of this study, fluoroquinolone use has also been widely reported in cats, both overall and for lower urinary tract disease.^1,10^ While excellent antimicrobials for bacterial urinary tract disease, fluoroquinolones are not recommended first-choice treatments for cystitis.^4,5^ Use of the third-generation cephalosporin and fluoroquinolone drug classes is under scrutiny in veterinary medicine, and further study of reasons for use of cefovecin, fluoroquinolones and HP-CIAs, in general, is required. Some use is indicated based on an inability to treat the cat orally (ie, cefovecin) or because of a need for a once-daily treatment regimen (eg, fluoroquinolones and cefpodoxime), but differentiating need from other reasons is required for assessment of the indications for use and development of effective interventions. Reasons for use of different treatment regimens could not be assessed in this study, but it is likely that convenience rather than need drives frequent use of cefovecin. This assumption is supported by the concurrent prescription of cefovecin and oral antimicrobials in a small percentage of cats, indicating that an inability to administer oral medications was not the reason for cefovecin use in those cases.

There has been limited study of duration of treatment of urinary tract disease in cats. A Swiss study reported a median duration of 10 days for the broad category of feline lower urinary tract disease,^10^ similar to the results of this study. A median of 10 days was prescribed when oral medications were used, but the frequent prescription of cefovecin resulted in a median duration of 14 days. The 10-day duration is consistent with 2011 ISCAID guidelines^4^ but higher than the 3–5-day duration recommended most recently.^5^ Shorter durations can be beneficial from many standpoints, including adverse event risks, stress of handling and pilling (to both cat and owner), and reduced antimicrobial resistance selection pressure. Short durations facilitate the use of oral treatments by reducing the amount of effort that is required for oral treatment. Owner preference (vs need) should not be the deciding factor when choosing a short course of a lower-tier drug vs an injectable third-generation cephalosporin.

Numerous differences were identified in prescriptions between different clinic types, with greater use of oral treatments (especially amoxicillin–clavulanic acid) and shorter prescribed durations at specialty clinics. The different nature of caseloads between clinic types must be considered; however, it would be reasonable to expect that specialty clinics would deal with more complex or compromised cases, biasing toward longer durations or use of broader spectrum antimicrobials, not the opposite. Clinics that have both primary and specialty care had results that were subjective intermediary between primary care and specialty clinics. This is unsurprising but perhaps supports the apparent differences between approaches in primary and specialty care. Reasons for this are unclear and could not be investigated with these data, but could include greater awareness of guidelines, greater confidence changing prescribing behaviors and differences in client interactions (eg, greater tendency to discuss or recommend oral administration).

The differences in treatment duration and drug selection (particularly the use of cefovecin) between prescribing in Canada and the USA were interesting. Reasons for differences between prescribing practices in Canadian and US clinics deserve further investigation and could relate to differences in veterinary education, continuing education, awareness of antimicrobial stewardship, awareness of guidelines and perceptions about clients’ expectations.

From an adverse event risk standpoint, it was encouraging to observe limited use of enrofloxacin vs other fluoroquinolones, based on safety issues (ie, retinopathy) that can be associated with use of enrofloxacin, but not other fluoroquinolones, in cats.^12^ However, there is still room for improvement, both in terms of the frequency of use of fluoroquinolones overall and the use of enrofloxacin.

The decrease in HP-CIA use from 2016 to 2018 for sporadic bacterial cystitis noted here was driven by reductions of cefovecin, and is encouraging, though there was an increase in fluoroquinolone use that raises concern. This likely reflects some changes from cefovecin to fluoroquinolones, and highlights the potential for further improvement by changing from oral fluoroquinolones to lower-tier options (ideally amoxicillin).

Medical records-based studies such as this have many inherent limitations based on gaps in the available data. Factors that might have influenced antimicrobial choices were not typically available, apart from text in the diagnostic field box. While guidelines provide good guidance, they cannot indicate what should be done in all cases, and individual patient factors can result in logical deviations in drug or duration choices from existing guidelines. Culture data were also not available. Anecdotally, bacterial culture is only performed in a minority of cases, particularly sporadic cystitis. Lack of culture data has some impact on the assessment of drug choice. However, antimicrobials were prescribed at the time of examination, when culture results were not available.

Assigning a duration for cefovecin use in surveillance studies can be challenging. Here, 14 days was chosen based on previously published recommendations,^13^ an earlier comparative study of lower urinary tract infection in cats where 14 days of cephalexin was used as the comparison with cefovecin^14^ and the recommendation of a redosing interval of 7–14 days in the 2019 ISCAID guidelines.^5^

Overuse of antimicrobials in cats with non-infectious urinary tract disease (eg, feline idiopathic cystitis) is likely common^10,15^ and probably accounted for many (if not a substantial proportion of) cases in this data set. However, the objectives of this study were to assess antimicrobial use practices when a veterinarian had decided to treat. Therefore, the inclusion of cats that did not actually have an infection or that were misclassified (eg, diagnosed as pyelonephritis when they actually had lower urinary tract disease or non-infectious disease) does not impact assessment of clinician treatment choices. Similarly, some cases were reported as ‘possible’ or ‘suspected’ infections but were included because an antimicrobial was prescribed. Changes in diagnoses made after the results of diagnostic testing were also not identifiable. This could have influenced duration, if antimicrobial courses were extended based on testing results. However, it still reflects how antimicrobials were initially prescribed, even if the reasons were not clear. There was a need to interpret disease categorization in some situations. While there were likely some errors in categorization, it is assumed that they represented a small percentage of cases. If these resulted in outliers, the non-parametric analyses would limit their impact.

## Conclusions

Understanding how antimicrobials are used is a key component of antimicrobial stewardship and is required to establish benchmarks, identify areas for improvement, aid in the development of interventions and to evaluate the impact of interventions or other changes.
